# Room Temperature Processed Double Electron Transport Layers for Efficient Perovskite Solar Cells

**DOI:** 10.3390/nano11020329

**Published:** 2021-01-27

**Authors:** Wen Huang, Rui Zhang, Xuwen Xia, Parker Steichen, Nanjing Liu, Jianping Yang, Liang Chu, Xing’ao Li

**Affiliations:** 1 New Energy Technology Engineering Laboratory of Jiangsu Provence and School of Science, Nanjing University of Posts and Telecommunications (NUPT), 9 Weiyuan Road, Nanjing 210023, China; wenhuang@njupt.edu.cn (W.H.); 1020082232@njupt.edu.cn (X.X.); 2 Key Laboratory for Organic Electronics and Information Displays and Institute of Advanced Materials, Jiangsu National Synergistic Innovation Center for Advanced Materials, School of Materials Science and Engineering, Nanjing University of Posts and Telecommunications (NUPT), 9 Wenyuan Road, Nanjing 210023, China; 15005187656@163.com (R.Z.); 17712910063@163.com (N.L.); yangjp@njupt.edu.cn (J.Y.); 3 Department of Materials Science and Engineering, University of Washington, Seattle, WA 98195-2120, USA; parker7s@uw.edu

**Keywords:** perovskite, interface, double electron transport layer, energy band matching, electron transport

## Abstract

Zinc Oxide (ZnO) has been regarded as a promising electron transport layer (ETL) in perovskite solar cells (PSCs) owing to its high electron mobility. However, the acid-nonresistance of ZnO could destroy organic-inorganic hybrid halide perovskite such as methylammonium lead triiodide (MAPbI_3_) in PSCs, resulting in poor power conversion efficiency (PCE). It is demonstrated in this work that Nb_2_O_5_/ZnO films were deposited at room temperature with RF magnetron sputtering and were successfully used as double electron transport layers (DETL) in PSCs due to the energy band matching between Nb_2_O_5_ and MAPbI_3_ as well as ZnO. In addition, the insertion of Nb_2_O_5_ between ZnO and MAPbI_3_ facilitated the stability of the perovskite film. A systematic investigation of the ZnO deposition time on the PCE has been carried out. A deposition time of five minutes achieved a ZnO layer in the PSCs with the highest power conversion efficiency of up to 13.8%. This excellent photovoltaic property was caused by the excellent light absorption property of the high-quality perovskite film and a fast electron extraction at the perovskite/DETL interface.

## 1. Introduction

Organic-inorganic hybrid halide perovskites CH_3_NH_3_PbX_3_ (X=I, Br, or Cl) are very promising materials in perovskite solar cells (PSCs) owning to their tunable direct bandgap [1], high light absorption coefficient [2], excellent carrier mobility [3] and long carrier diffusion length [4]. However, there are a few limitations for the applications of these PSCs [5]. For example, the perovskite materials can break down due to the influence of the ambient environment such as heat, moisture and nearby materials [6,7]. The preparation of the electron transport layer (ETL) in PSCs when using materials such as TiO_2_ requires high temperature annealing [8]. This has prevented the development of PSCs as a promising future clean energy. In recent years, many researchers have worked on PSCs to solve these challenges [9,10,11,12].

**Figure 1:**
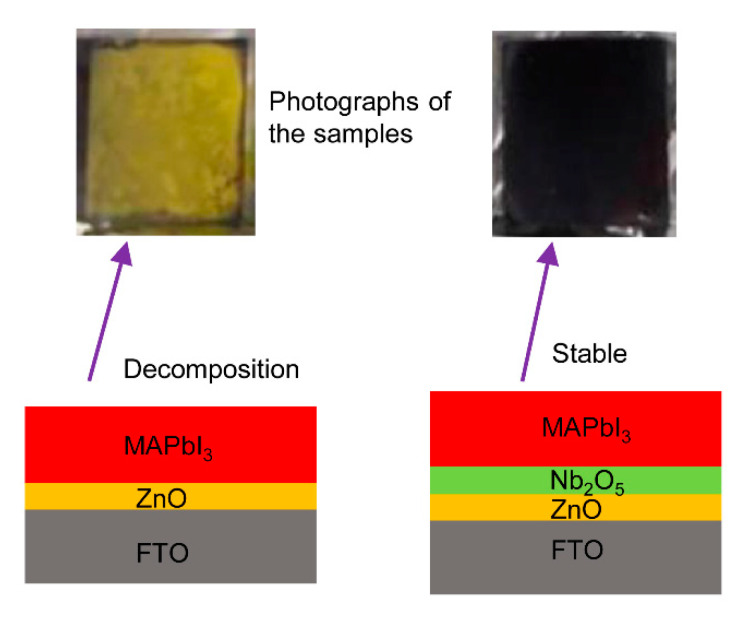
Photographs of MAPbI_3_ film on ZnO (**left**) and Nb_2_O_5_/ZnO (**right**).

The PSCs usually have “p-i-n(n-i-p)” type planar sandwiched structures, where “p” is the hole transport layer (HTL), “i” is the intrinsic light absorption layer (perovskite) and “n” is the ETL [13,14]. There are many candidate materials such as TiO_2_ and ZnO for the ETL in PSCs [15,16,17,18]. By contrast, the temperature required for the deposition of ZnO for the ETL is notably lower than that of a TiO_2_ mesoporous film. Furthermore, the electron mobility of ZnO is substantially higher than TiO_2_. These advantages make ZnO an ideal choice for the ETL [19]. However, studies have effectively demonstrated that organic-inorganic hybrid halide perovskite MAPbI_3_ may be degraded into PbI_2_ if MAPbI_3_ is directly deposited on ZnO, accompanied by the appearing of a yellow color [20,21,22]. This phenomenon was also found in the current work as shown in the left part of Figure 1. The MAPbI_3_ film decomposed into a yellow-colored byproduct for the case of MAPbI_3_/ZnO. This was caused by the basic nature of the ZnO surface [20], which led to deprotonation of the methylammonium cation and the formation of PbI_2_. The process could be accelerated by the presence of surface hydroxyl groups and/or residual acetate ligands. The efficiency was then expected to be extremely low or zero after the decomposition of MAPbI_3_ in the PSCs. To overcome this drawback, Cao et al. [23] modified the surface of ZnO using MgO and ethanolamine in the PSCs. This improved the performance of the PSCs while the energy band of MgO and ZnO as well as perovskite was not matched. Zuo and co-workers [24] deposited 3-aminopropanoic acid SAM (C3-SAM) onto sol−gel ZnO layers and induced a significant improvement in the morphology of the perovskite film. However, the annealing temperature for the ZnO film of 160 °C in this spin-coating method limited its application in many areas. 

Previously, Nb_2_O_5_ was used to modify ZnO in the study of dye-sensitized solar cells and exhibited compatibility between Nb_2_O_5_ and ZnO [25]. This indicated the possibility of using Nb_2_O_5_ /ZnO as the double electron transport layer (DETL) in MAPbI_3_-based solar cells. Firstly, Nb_2_O_5_ can be prepared by many methods at room temperature with low cost and through simple processes [26,27,28,29]. Secondly, the conduction band of Nb_2_O_5_ is between ZnO and MAPbI_3_ [30,31], which enables the rapid injection of electrons from the perovskite layer into the ZnO and bottom electrode. Furthermore, the insertion of Nb_2_O_5_ can prevent the chemical decomposition of MAPbI_3_ caused by ZnO. MAPbI_3_ films deposited directly onto a Nb_2_O_5_/ZnO DETL showed no changes in its color (dark brown) as shown in the right part of Figure 1. This indicated that the insertion of Nb_2_O_5_ prevented chemical decomposition and improved the stability of the perovskite film. This finding motivated our study of ultra-thin Nb_2_O_5_/ZnO films as a promising DETL used in PSCs.

With the above consideration, in this work we deposited Nb_2_O_5_/ZnO thin films at room temperature with a magnetron sputtering technique to create efficient and stable PSCs [32]. In the fabricated solar cells, Nb_2_O_5_/ZnO thin films were adopted as the DETL. The PCE of PSCs based on Nb_2_O_5_/5-ZnO (a ZnO deposition time of five minutes) thin film was found to reach the highest efficiency of 13.8%. The room temperature processing and relatively high device performance suggest great potential for Nb_2_O_5_/ZnO thin films as the DETL in applications such as large area solar cells and other optoelectrical devices.

## 2. Experimental Section

### 2.1. Materials Preparation 

All of the solvents were purchased from Sigma-Aldrich. MAI (99.99%), PbI_2_ (99.99%) and Spiro-OMeTAD (99.8%) were purchased from Xi’an Polymer Light Technology Corp (Xi’an, China). Nb_2_O_5_ and ZnO target materials were purchased from Hebei Qinbang New Material Technology Co. Ltd (Handan, China). Fluorine tin oxide (FTO) glass was purchased from Nippin Sheet Glass Co. Ltd (Minato, Japan). A MAPbI_3_ precursor solution was formed through dissolving 159 mg methyl ammonium iodine (MAI) and 480 mg PbI_2_ in 800 μL of a solution of dimethyl sulfoxide (DMSO) and *N*, *N*-dimethylformamide (DMF) (volume ratio of DMSO to DMF was 1:4). The precursor solution of Spiro-OMeTAD for the hole transport layer was formed by dissolving 72.3 mg Spiro-OMeTAD in 1 mL chlorobenzene. In sequence, 17.5 μL acetonitrile solution of Lithium bis(trifluoromethylsulphonyl)imide (LiTFSI) (520 mg/mL) and 28 μL 4-tert-butylpyridine were added to the resulting solution.

### 2.2. Deposition of Nb_2_O_5_/ZnO Films

ZnO thin films were deposited at room temperature by RF magnetron sputtering in an argon (99.999%) atmosphere using a pure ZnO target (99.99%). Initially, the vacuum was pumped to 10^−4^ Pa and the target was exposed to pure Ar and oxygen gas (purity 99.999%) with a flow rate of 60 sccm and 1 sccm, respectively. This led to a chamber pressure of 0.5 Pa. The distance between the substrate and ZnO target was 20 cm and the sputtering power was set to 60 W. The substrate rotation speed was 2 rad/s. A baffle plate over the ZnO target was closed and the target was pre-sputtered for four minutes to remove dust and impurities. ZnO films were then deposited by opening the baffle plate to expose the substrate to the target for a set time before again closing the baffle. The films’ thickness was varied under a deposition time of three, five and eight minutes, respectively.

Nb_2_O_5_ thin films were prepared at room temperature with RF magnetic sputtering in an argon (99.999%) atmosphere using a pure Nb_2_O_5_ target (99.999%). Before sputtering, the vacuum was pumped to 10^−4^ Pa. The target was then exposed to pure argon and oxygen gas with a flow rate of 20 sccm, leading a chamber pressure of 0.3 Pa. The distance between the substrate and the Nb_2_O_5_ target was 20 cm and the sputtering power was 50 W. The substrate rotation speed was 2 rad/s. The baffle plate of Nb_2_O_5_ target was closed in the beginning and the target was pre-sputtered for four minutes to remove dust and impurities. The substrate baffle of the target was then opened and an ultra-thin Nb_2_O_5_ film was obtained after two minutes’ sputtering. Finally, Nb_2_O_5_/ZnO DETLs were formed.

### 2.3. Device Fabrication

The fluorine-doped tin oxide (FTO) glass substrates (1.45 cm × 1.45 cm) were firstly ultra-sonically cleaned with deionized water, acetone and ethanol for 15 minutes, respectively. These substrates were then treated with UV-ozone for 20 minutes. The Nb_2_O_5_/ZnO thin films for DLET were deposited onto the substrates by the method described above. A MAPbI_3_ precursor solution was subsequently spin-coated on the ETL to form perovskite MAPbI_3_ films through a one step process, which included two-speed steps (i.e., 500 rpm for 3 s followed by 4000 rpm for 20 s). During the second step, about 300 μL of chlorobenzene was added by dropping after 10 s spin-coating. The device was then annealed in an N_2_ atmosphere at 75 °C for 10 minutes followed by a second 10 minutes’ annealing at 105 °C. After annealing, a Spiro-OMeTAD solution was spin-coated at 3000 rpm for 30 s. Finally, 150 nm thick silver electrodes were prepared by thermal evaporation under a 10^−5^ bar vacuum condition.

### 2.4. Characterization

Elements of Zn and Nb were verified using the X-ray photoelectron spectroscopy analyzer (AXIS Supra) with a monochromatic Al Kα X-ray source. Surface morphologies of the perovskite films were measured by scanning electron microscopy (SEM, FEI NOVA Nano SEM 450). X-ray diffraction (XRD) patterns were analyzed by an X-ray diffractometer (Bruker D8 Advance, Ettlingen, Germany) with a Cu-Kα radiation source (λ=1.5418 Å). The absorption spectra were measured with an UV-Vis spectrophotometer (PerkinElmer Lambda 650 S, Nanjing, China) in the range from 450 to 800 nm. The current voltage (J-V) characteristics were obtained at a solar simulator (AM 1.5 G, 100mWcm^−2^, Newport 91150, USA) equipped with a Keithley 2400 source meter. The incident photon-to-electron conversion efficiency (IPCE) of the devices was characterized on a computer-controlled IPCE system (Newport). The electrochemical impedance spectroscopy (EIS) was measured under a positive bias of 1 V, with an amplitude of 0.01 V and a frequency range from 1 Hz to 1 MHz. The photoluminescence spectra (excitation at 485 nm) were recorded by an Edinburgh F900.

## 3. Results and Discussion

**Figure 2:**
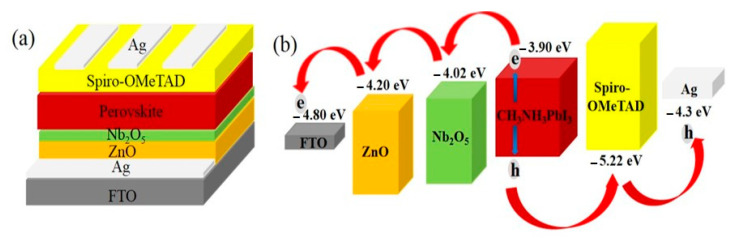
(**a**) Schematics of the planar perovskite solar cells (PSCs) based on an Nb_2_O_5_/ZnO thin film, (**b**) Energy band alignment of the devices.

The planar PSCs with a DETL were fabricated as shown in Figure 2a. The schematic of the energy band alignment is shown in Figure 2b. In the fabricated PSCs, the electrode FTO was used as the bottom cathode and Spiro-OMeTAD and Ag as the HTL and top anode, respectively. MAPbI_3_ was used as the optically active layer. ZnO was used as the ETL. The ZnO/perovskite interface was mediated by a thin layer of Nb_2_O_5_. Such an interfacial structure plays two important roles: (1) the Nb_2_O_5_ film stopped the MAPbI_3_ from reacting with the ZnO. This was confirmed visually in Figure 1. (2) The ultra-thin Nb_2_O_5_ film was beneficial to the electron transport at the interfaces due to the matching of the energy band structure as shown in Figure 2b [33,34,35]. Thus, Nb_2_O_5_/ZnO films were used as the DETL in this study. The carrier transport process in the device is explained by the following process. Electron hole pairs were generated in response to external light exposure. Under the driving force of the built-in electric field at the interfaces between perovskite and transport layers, the holes were transferred into the Ag electrode through Spiro-OMeTAD while electrons were rapidly injected into the Nb_2_O_5_/ZnO layers from the MAPbI_3_ [34].

**Figure 3:**
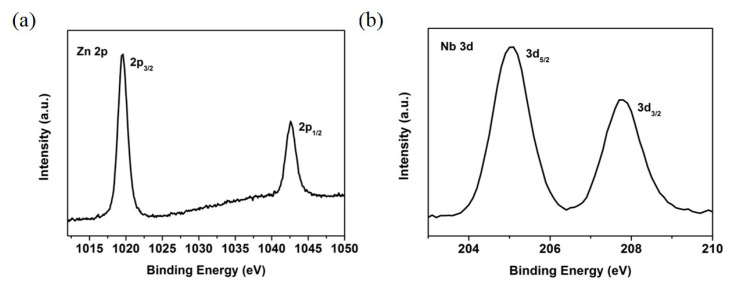
(**a**) Zn 2p high resolution XPS spectrum of ZnO thin film; (**b**) Nb 3d high resolution XPS spectrum of Nb_2_O_5_/ZnO thin film.

The double Nb_2_O_5_/ZnO electron transport layers were deposited on an FTO glass substrate with magnetron sputtering. During this process, the films’ thicknesses could be obtained using a quartz crystal thickness monitor [36]. Considering the accuracy limitations of the quartz crystal thickness monitor in determining the ultra-thin films in the current work, the thickness of the ZnO and Nb_2_O_5_ films was then determined, respectively, based on the obtained deposition rates by controlling the deposition times. The deposition rates of these films with current experimental procedures were previously estimated in the same lab. In the current work, the deposition time for Nb_2_O_5_ was five minutes and the thickness was then estimated to be ~15 nm. The deposition time of the ZnO films was three minutes (Nb_2_O_5_/3-ZnO), five minutes (Nb_2_O_5_/5-ZnO) and eight minutes (Nb_2_O_5_/8-ZnO), respectively. Their thicknesses corresponded to 12 nm, 20 nm and 32 nm accordingly. This is similar to the way of, for example, Nb-doped TiO_2−*x*_ film (used as an electrode and ETL) thickness control through adjusting the deposition time in a perovskite solar cell in the work of Kim et al. [37]. However, the film thickness (300 nm) was measured using a transmission electron microscope technique in their work. To confirm the presence of Nb_2_O_5_ and ZnO films on the FTO, the electronic states of the deposited ZnO and Nb_2_O_5_/ZnO films were characterized by XPS spectra. Figure 3a shows a high resolution XPS spectrum of the Zn 2p in the ZnO thin film. It can be clearly seen that there were two peaks located at the binding energies of 1019.5 eV and 1042.5 eV, which corresponded to Zn 2p_3/2_ and Zn 2p_1/2_, indicating the existence of divalent zinc. This meant the ZnO thin film was successfully covered on the FTO glass substrates. Figure 3b shows the high resolution XPS spectrum of Nb 3d in the Nb_2_O_5_/ZnO thin film. It shows double peaks at the positions of binding energy of 205.0 eV and 207.7 eV, corresponding to Nb 3d_5/2_ and Nb 3d_3/2_, respectively, demonstrating the existence of pentavalent niobium. This confirmed that ultra-thin Nb_2_O_5_ thin films were deposited on ZnO thin films completely. Therefore, these findings indicated that an Nb_2_O_5_/ZnO DETL was successfully deposited on the glass substrate. 

**Figure 4:**
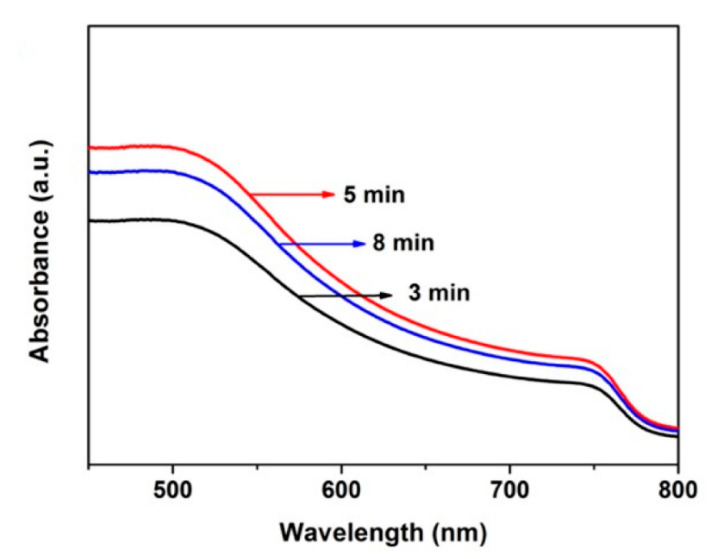
UV-Vis absorption spectra of perovskite films deposited on Nb_2_O_5_/3-ZnO (3 min), Nb_2_O_5_/5-ZnO (5 min) and Nb_2_O_5_/8-ZnO (8 min), respectively.

The MAPbI_3_ film was spin-coated onto the DETL. The optical absorption properties of the perovskite film were investigated using the UV-Vis spectroscopy. The results are presented in Figure 4. The perovskite film on Nb_2_O_5_/5-ZnO revealed a stronger light absorption property than that of Nb_2_O_5_/8-ZnO. The absorption property was the worst in the film based on Nb_2_O_5_/3-ZnO. This trend could be related to the improvement of the crystallizing quality of the perovskite film based on the different ETLs in the order of Nb_2_O_5_/3-ZnO→Nb_2_O_5_/8-ZnO→Nb_2_O_5_/5-ZnO from the XRD patters (Figure S1). The bandgap of ZnO was around 3.2 eV. This indicated that a thin ZnO film was transparent to the wavelength of light larger than 387 nm and could be excluded for the causes of observed difference of light absorption [38].

**Figure 5:**
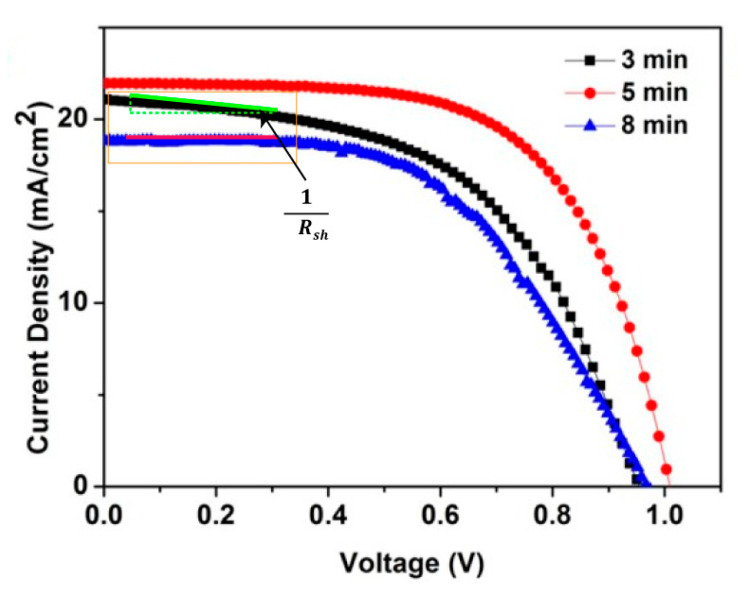
J-V curves of the solar cells based on Nb_2_O_5_/3-ZnO (3 min), Nb_2_O_5_/5-ZnO (5 min) and Nb_2_O_5_/8-ZnO (8 min) under light illumination.

**Figure 6:**
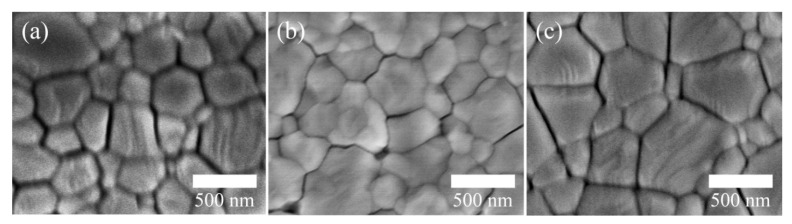
SEM of perovskite films deposited on (**a**) Nb_2_O_5_/3-ZnO (3 min), (**b**) Nb_2_O_5_/5-ZnO (5 min) and (**c**) Nb_2_O_5_/8-ZnO (8 min).

The current density versus voltage (J-V) curves of the PSCs based on Nb_2_O_5_/3-ZnO, Nb_2_O_5_/5-ZnO and Nb_2_O_5_/8-ZnO were measured under 100 mW/cm^2^ (AM 1.5G) light illumination by a sunlight simulator, as shown in Figure 5. The photovoltaic performances of the PSC devices (open-circuit voltage *V*_oc_, short-circuit current *J*_sc_, fill factor FF and PCE) are summarized in Table 1. It was shown that Nb_2_O_5_/5-ZnO-based PSCs showed the best photovoltaic performance. The morphology and surface coverage of the perovskite thin films were characterized using SEM to further verify the quality of the film. The results are shown in Figure 6. The perovskite film based on Nb_2_O_5_/5-ZnO exhibited a well-connected morphology in which the cracks between the grain boundaries were obviously reduced compared with those on Nb_2_O_5_/8-ZnO and Nb_2_O_5_/3-ZnO. This could inhibit the interface recombination of carriers in these regions and contribute to the observed excellent properties based on Nb_2_O_5_/5-ZnO.

In order to verify the accuracy of *J*_SC_ in Table 1, the IPCE on these devices was measured. The theoretical values of *J*_SC_ were extracted from the spectra as shown in Figure S2. The *J*_SC_ was 20.3 mA/cm^2^, 21.2 mA/cm^2^ and 18.1 mA/cm^2^ based on Nb_2_O_5_/3-ZnO, Nb_2_O_5_/5-ZnO and Nb_2_O_5_/8-ZnO, respectively. It was consistent with the results of the J-V curves, thereby successfully verifying the accuracy of experimental results. Although the absorption property of the perovskite in the case of Nb_2_O_5_/3-ZnO was the worst, the thinner Nb_2_O_5_/3-ZnO could lead to less recombination of the carriers and offset a lower light absorption of the perovskite. Therefore, *J*_SC_ and the IPCE in the case of Nb_2_O_5_/3-ZnO and Nb_2_O_5_/5-ZnO are close.

**Figure 7:**
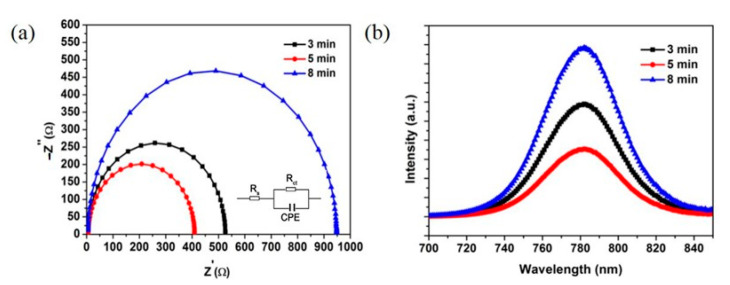
(**a**) Electrochemical impedance spectroscopy (EIS) Nyquist plots (the inset is the equivalent circuit) based on Nb_2_O_5_/3-ZnO (3 min), Nb_2_O_5_/5-ZnO (5 min) and Nb_2_O_5_/8-ZnO (8 min). (**b**) Photoluminescence (PL) spectra of the PSCs based on Nb_2_O_5_/3-ZnO (3 min), Nb_2_O_5_/5-ZnO (5 min) and Nb_2_O_5_/8-ZnO (8 min).

Electrical impedance spectroscopy (EIS) was employed to examine carrier transfer at the interfaces of the perovskite and different ETLs under dark conditions so as to further understand the influence of different ETLs on the photovoltaic properties. Figure 7a shows a Nyquist diagram of PSCs based on Nb_2_O_5_/3-ZnO, Nb_2_O_5_/5-ZnO and Nb_2_O_5_/8-ZnO, which were measured under a 1 V forward bias. The equivalent circuit model is shown in the insert of Figure 7a and is composed of solution resistance (R_s_) and charge transfer resistance (R_ct_). On account of the semblable device structure of the PSCs, the solution resistance (R_s_) was almost the same. Three separate semicircles in Nyquist plots were obtained by frequency analysis; the radius of the semicircles represents R_ct_ [39], which could be associated with the perovskite/Nb_2_O_5_/ZnO interfaces. Apparently, the R_ct_ of the device based on Nb_2_O_5_/5-ZnO was smaller than that obtained from the devices based on Nb_2_O_5_/3-ZnO and Nb_2_O_5_/8-ZnO. This indicated that the PSCs based on Nb_2_O_5_/5-ZnO had a greater charge collection and transport abilities at the interfaces than the others. To further verify this, steady-state photoluminescence (PL) spectra were used to explore the charge transfer kinetics at the perovskite/Nb_2_O_5_/ZnO interfaces as shown in Figure 7b. The PL peaks around 770 nm were attributed to the emission from the MAPbI_3_ [40]. More significant emission quenching was clearly observed for the device based on Nb_2_O_5_/5-ZnO compared with the devices based on Nb_2_O_5_/3-ZnO and Nb_2_O_5_/8-ZnO. These confirmed that the electron transfer through the perovskite/Nb_2_O_5_/5-ZnO interface was faster and more effective, which contributed to the best photovoltaic properties. These properties were related to the compact perovskite film and less recombination centers at the interfaces based on Nb_2_O_5_/5-ZnO. 

We would like to mention that although the R_ct_ value of Nb_2_O_5_/8-ZnO was larger than that of Nb_2_O_5_/3-ZnO, the fill factor (FF) values were close for Nb_2_O_5_/3-ZnO and Nb_2_O_5_/8-ZnO. Based on FF = FF_0_ (1-R_se_/R_sh_) [41], R_se_ was the series resistance and R_sh_ the shunt resistance. R_se_ was approximately regarded as the charge transfer resistance R_ct_. This indicated that R_sh_ for Nb_2_O_5_/8-ZnO was larger than Nb_2_O_5_/3-ZnO. This could be proved from the J-V curves in Figure 5, in which R_sh_ for Nb_2_O_5_/8-ZnO was larger than Nb_2_O_5_/3-ZnO extracted from the slope (1/R_sh_) near the short-circuit current point.

It is interesting to observe that the perovskite film quality that influenced the PCEs relied on the deposition time of ZnO (ETL) in the DETLs. The influence of ETL thickness on the PCE was found in a perovskite solar cell based on a Nb-doped TiO_2−*x*_ film as the ETL as well [37]. Kim et al. argued that the obtained best PCE could be related to the excellent electrical properties of the Nb-doped TiO_2−*x*_ film for the optimized thickness. We would like to mention that the PCE of the perovskite solar cell based on Nb_2_O_5_/5-ZnO in the current work was still much lower than that of other reported perovskite solar cells [41]. This indicated that the quality of Nb_2_O_5_/5-ZnO may still not fulfill the high-quality requirement for an electron transport layer in the perovskite solar cell. For example, a serious recombination of carriers may still exist in Nb_2_O_5_/5-ZnO or the interfaces between Nb_2_O_5_/5-ZnO and MAPbI_3_ if the crystallization was not excellent. A rough morphology of Nb_2_O_5_/5-ZnO may influence the crystalline quality of MAPbI_3_ and then lead to weak light absorption properties. The best quality perovskite film obtained on a DETL could be related to more complete crystallization of the DETL. This process involves the growth kinetics of thin films and the corresponding deposition procedures (MAPbI_3_ and DETL) and mechanisms that call for further investigation. The influence of Nb_2_O_5_ thickness on the PCE of the PSCs is expected and therefore further investigations are needed to optimize the thickness of this layer. This would significantly deepen our understanding of DETL effects on PCE and promote the application of Nb_2_O_5_/ZnO films in PSCs.

## 4. Conclusions

In summary, we have demonstrated room temperature processed Nb_2_O_5_/ZnO thin films as the DETL used in MAPbI_3_-based PSCs. The planer PSCs based on Nb_2_O_5_/5-ZnO achieved a PCE of 13.8%. This high device performance was attributed to excellent high-quality perovskite film and a perovskite/ETL interface based on Nb_2_O_5_/5-ZnO film. In addition, the insertion of Nb_2_O_5_ in the current work prevented the chemical decomposition of MAPbI_3_ caused by contact with ZnO. The improved chemical stability, energy band matching and room temperature processing with relatively high device performance suggested great potential for a DETL of Nb_2_O_5_/ZnO in large area solar cells and other optoelectrical devices.

## Figures and Tables

**Table 1:** Performance parameters of PSCs based on Nb_2_O_5_ / ZnO films.

Sputter ZnO Time (min)	V_OC_ (V)	J_SC_ (mA/cm^2^)	FF (%)	PCE (%)
3	0.94	21.0	52.4	10.5
5	1.0	21.9	62.7	13.8
8	0.97	18.9	53	9.7

## Supplementary Materials

The following are available online at https://www.mdpi.com/2079-4991/11/2/329/s1, Figure S1: XRD patters of perovskite films based on Nb_2_O_5_/3-ZnO, Nb_2_O_5_/5-ZnO and Nb_2_O_5_/8- ZnO, respectively, Figure S2: IPCE spectra and current density of the PSCs based on Nb_2_O_5_/3-ZnO, Nb_2_O_5_/5-ZnO and Nb_2_O_5_/8-ZnO, respectively.
