# Supplementary material for: Room Temperature Processed Double Electron Transport Layers for Efficient Perovskite Solar Cells
Source: Nanomaterials (Basel). 2021 Jan 27;11(2):329. doi: 10.3390/nano11020329 (PMC7911782; doi:10.3390/nano11020329)
Supplement: Supplementary file 1 [file nanomaterials-11-00329-s001.pdf]

Supplementary Material

Room Temperature Processed Double Electron Transport Layers for Efficient Perovskite Solar Cells

Wen Huang [1], Rui Zhang [2], Xuwen Xia [1], Parker Steichen [3], Nanjing Liu [2], Jianping Yang [2], Liang Chu [1,2,*] and Xing'ao Li [1,2,*]

[1] New Energy Technology Engineering Laboratory of Jiangsu Provence and School of Science, Nanjing University of Posts and Telecommunications (NUPT), 9 Weiyuan Road, Nanjing 210023, China; wenhuang@njupt.edu.cn (W.H.); 15005187656@163.com (X.X.)
[2] Key Laboratory for Organic Electronics and Information Displays and Institute of Advanced Materials, Jiangsu National Synergistic Innovation Center for Advanced Materials, School of Materials Science and Engineering, Nanjing University of Posts and Telecommunications (NUPT), 9 Wenyuan Road, Nanjing 210023, China; 15005187656@163.com (R.Z.); 17712910063@163.com (N.L.); yangjp@njupt.edu.cn (J.Y.)
[3] Department of Materials Science and Engineering, University of Washington, Seattle, WA 98195-2120, USA; parker7s@uw.edu
* Correspondence: chuliang@njupt.edu.cn (L.C.); lixa@njupt.edu.cn (X.L.)

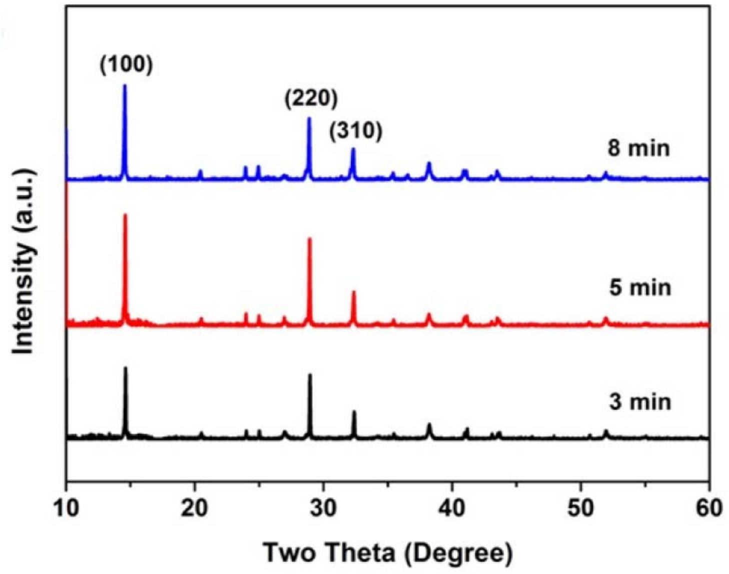

Figure S1. XRD patters of perovskite films based on Nb_2O_5/3-ZnO, Nb_2O_5/5-ZnO and Nb_2O_5/8-ZnO, respectively.

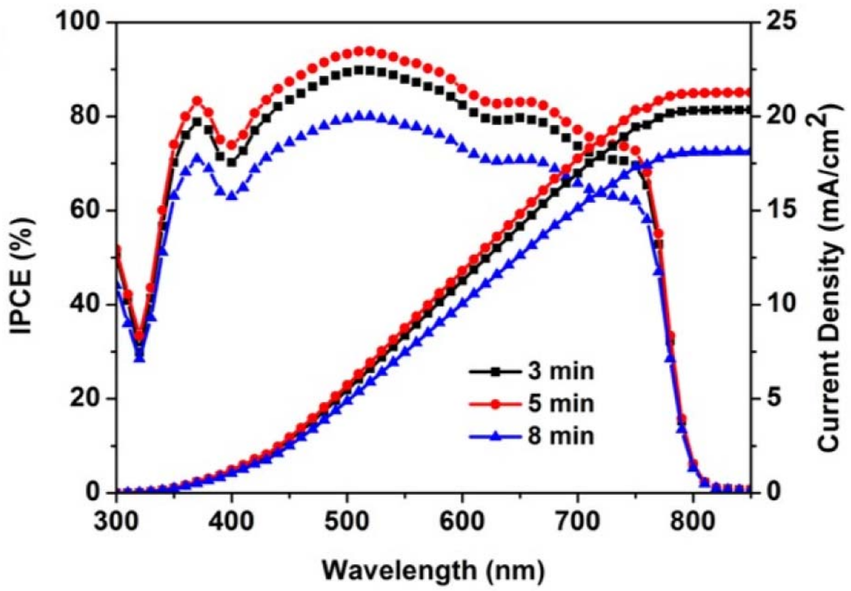

Figure S2. IPCE spectra and current density of the PSCs based on Nb_2O_5/3-ZnO, Nb_2O_5/5-ZnO and Nb_2O_5/8-ZnO, respectively.
